# Einfaches Verfahren zur Abschätzung des postoperativen Abbildungsmaßstabs und der Aniseikonie bei der Kataraktoperation

**DOI:** 10.1007/s00347-021-01412-6

**Published:** 2021-06-01

**Authors:** Achim Langenbucher, Peter Hoffmann, Jascha Wendelstein, Nóra Szentmáry

**Affiliations:** 1 Institut für Experimentelle Ophthalmologie, Universität des Saarlandes, Kirrberger Str., Gebäude 22, 66421 Homburg, Deutschland; 2 Augen- und Laserklinik Castrop-Rauxel, Castrop-Rauxel, Deutschland; 3 Klinik für Augenheilkunde und Optometrie, Johannes Kepler Universität Linz, Linz, Österreich; 4 Dr. Rolf M. Schwiete Zentrum für Limbusstammzellforschung und kongenitale Aniridie, Universität des Saarlandes, 66421 Homburg, Deutschland; 5 Universitäts-Augenklinik, Semmelweis-Universität, Budapest, Ungarn

**Keywords:** Aniseikonie, Retinale Bildgröße, Abbildungsmaßstab, Biometrie, Linsenberechnung, Aniseikonia, Retinal image size, Ocular magnification, Biometry, Lens power calculation

## Abstract

**Hintergrund und Zielsetzung:**

Die Aniseikonie als mögliche Ursache asthenopischer Beschwerden tritt bei der modernen Kataraktchirurgie oft in den Hintergrund. Ziel der vorliegenden Arbeit ist es, dem Kliniker ein einfaches Berechnungsmodell an die Hand zu geben, mit dem der Abbildungsmaßstab des pseudophaken Auges abgeschätzt werden kann.

**Methoden:**

Das Berechnungsschema für den Abbildungsmaßstab des pseudophaken Auges bezieht sich auf die formelbasierte (vergenzbasierte) Berechnung der Intraokularlinse mit theoretisch-optischen Formeln. Aus den biometrischen Größen, die in der Regel für beide Augen bei der Linsenberechnung vorliegen, kann aus den Vergenzen vor und hinter den 3 oder 4 refraktiven Grenzflächen im pseudophaken Augenmodell der Abbildungsmaßstab für Objekte im Unendlichen oder in einer endlichen Messdistanz ermittelt werden.

**Ergebnisse:**

Bei der formelbasierten Berechnung wird ein pseudophakes Augenmodell mit 3 bzw. 4 refraktiven Grenzflächen (postoperative Brillenrefraktion; dünne Hornhaut, beschrieben durch die Vorderfläche, bzw. dicke Hornhaut, beschrieben durch die Vorder- und Rückfläche; Intraokularlinse) definiert und mit den Methoden der linearen Optik die Vergenz vor und hinter jeder Grenzfläche bestimmt. Der Quotient aus dem Produkt der Vergenzen vor den Grenzflächen und dem Produkt der Vergenzen unmittelbar hinter den Grenzflächen beschreibt direkt den Abbildungsmaßstab des Auges. Aus dem Vergleich des Abbildungsmaßstabs beider Augen kann unmittelbar der retinale Bildgrößenunterschied ermittelt werden. Exemplarisch wird dies anhand der Haigis- und Hoffer-Q-Formel (3 Flächen) und der Castrop-Formel (4 Flächen) gezeigt.

**Schlussfolgerungen:**

Wird bei der Planung der Kataraktoperation die Biometrie und Linsenberechnung an beiden Augen durchgeführt, so kann mit einfachen Mitteln der Abbildungsmaßstab bei beiden Augen und aus dem Vergleich beider Augen die Aniseikonie des pseudophaken Patienten ermittelt werden. Eine derartige Abschätzung sollte fester Bestandteil der Linsenberechnung werden, um mögliche asthenopische Beschwerden nach der Kataraktoperation früh zu erkennen.

## Abstract (en)

**Background and purpose:**

Aniseikonia as one of the major risk factors for asthenopic problems is mostly overlooked in modern cataract surgery. The purpose of this study was to develop a simple calculation scheme for clinicians to predict the object to image magnification in a pseudophakic eye with biometric data.

**Methods:**

The calculation scheme for object to image magnification in the pseudophakic eye is based on a vergence calculation of the lens power with theoretical optical formulae. From the biometric data, which are typically derived from both eyes during lens power calculation, the vergences in front of and behind the 3 or 4 refractive surfaces of the pseudophakic eye model are used to predict the magnification for objects at infinity or objects located at a finite measurement distance (e.g. 5 m).

**Results:**

With a formula-based lens power calculation a pseudophakic eye model is set up with 3 or 4 refractive surfaces (postoperative spectacle refraction; thick cornea described by anterior surface or thick cornea characterized by anterior and posterior surfaces; intraocular lens). The vergence in front of and behind each refractive surface is derived by means of linear Gaussian optics. The quotient of the product of all vergences in front of the surfaces and the product of all vergences behind the respective surfaces describes the object to image magnification of the eye. A comparison of the object to image magnification of both eyes yields the retinal image size disparity or aniseikonia. This calculation strategy is shown in a step-by-step approach exemplarily for the Haigis and Hoffer‑Q formulae (3 surfaces) and the Castrop formula (4 surfaces).

**Conclusion:**

If during planning and lens power calculation biometry is performed for both eyes, ocular magnification of both eyes can be easily derived with this calculation scheme and aniseikonia can be extracted from a comparison of magnification of both eyes. Such a simple prediction should be established as a standard for precataract biometry and lens power calculation for early detection and avoidance of asthenopic complaints after cataract surgery.

In der modernen Kataraktchirurgie steht dem Ophthalmochirurgen eine fast unüberschaubare Auswahl an Linsenimplantaten und -designs, aber auch an Berechnungsformeln oder Strategien zur Verfügung. Ausgehend von den Basisformeln von Fyodorov [1] oder Gernet, Ostholt und Werner [2], wurden in den 80er- und 90er-Jahren des vergangenen Jahrhunderts die „Klassiker“ wie die Haigis-Hoffer-Q- [3, 4], Holladay1- oder SRKT-Formel [18] entwickelt, die im Vergleich zur Regressionsformel SRK oder SRK2 von Sanders, Retzlaff und Kraff deutlich geringere Vorhersagefehler für die postoperative Refraktion v. a. für lange oder kurze Augen oder Augen mit flachen oder steilen Hornhautradien versprachen. Bei diesen Berechnungsformeln wurde ein „pseudophakes Augenmodell“ definiert, das mit biometrischen Messgrößen und ggf. daraus abgeleiteten Größen „gefüttert“ wird. All diese Formeln werden unter dem Begriff „Vergenzformeln“ subsumiert.

In den vergangenen 20 Jahren wurden dann sehr viele neue Linsenberechnungskonzepte entwickelt, die entweder auf empirischen Überlegungen, auf Kombinationen formaler Ansätze der Physik und der Empirie oder auf Raytracing-Strategien beruhen. Verfahren des maschinellen Lernens (z. B. Hill-RBF-Calculator) zählen hierbei zu den empirischen Ansätzen. Die meisten der neu entwickelten Berechnungsstrategien sind nicht oder nicht vollständig offengelegt, sodass die Formeln nicht oder nur unzureichend verglichen werden können.

Bei der Biometrie wurde zunächst mit der Einführung des optischen Biometers 1999 und in der Folge mit der Einführung der optischen Teilstreckenmessung ein Standard geschaffen, der in Kombination mit den Verbesserungen bei der Linsenberechnung einen hohen Standard bei der Linsenberechnung und der Vorhersage des refraktiven Ergebnisses gewährleistet. Unter Studienbedingungen kann heute erreicht werden, dass rund 65–80 % der Augen innerhalb eines Vorhersagefehlers der postoperativen Refraktion von ±0,5 dpt landen [12, 14, 17].

Allerdings wird heute die Bedeutung des retinalen Abbildungsmaßstabs bzw. die Aniseikonie als Maß für den retinalen Bildgrößenunterschied beider Augen weitgehend ignoriert [9]. Laut Literaturlage liegt der Bildgrößenunterschied bei phaken Augen in der Regel bei unter einem halben Prozent, allerdings können Bildgrößenunterschiede von über 10 % im Einzelfall auftreten [5, 6, 11]. Die Toleranz der Aniseikonie ist in der Bevölkerung sehr uneinheitlich: So kann im Einzelfall eine Aniseikonie von 2–3 % problemlos vom Patienten toleriert werden oder auch zu schneller Ermüdung beim Lesen führen. Bei 5–7 % Bildgrößenunterschied geht man im Allgemeinen von ernsten asthenopischen Beschwerden aus, und ab einer Aniseikonie von etwa 7 % ist die Fusion der beiden Netzhautbilder stark eingeschränkt oder gar unmöglich [15, 19–21]. Anisometropie als Seitenunterschied in der Bauform beider Augen oder der Refraktion ist generell die Ursache einer Aniseikonie, aber nicht jede Anisometropie führt zwangsläufig zu einer Aniseikonie [16]. So können mehrere Parameter mit Seitendifferenz beider Augen ihren Effekt auf die Aniseikonie entweder verstärken oder auch abschwächen.

Speziell bei einer anstehenden Kataraktoperation hat der Ophthalmologe die Möglichkeit, den postoperativen Abbildungsmaßstab beider Augen aus der Biometrie vorherzusagen und damit die Aniseikonie abzuschätzen. Sofern Vergenzformeln, wie z. B. die Haigis-Formel [18], die Olsen-Formel, Castrop-Formel oder die Hoffer-Q-Formel [3, 4], für die Berechnung der Stärke einer Intraokularlinse verwendet werden, kann der Abbildungsmaßstab aus dem Berechnungskonzept sehr einfach hergeleitet werden, da bei einer Implementierung in einem Berechnungsprogramm die benötigten Vergenzen vor und hinter den Grenzflächen entweder unmittelbar als Zwischenschritt der Berechnung vorliegen oder sehr einfach abgeleitet werden können.

*In dieser Studie soll aufgezeigt werden*, wie der Abbildungsmaßstab eines Auges bzw. die retinale Bildgröße im Rahmen einer Linsenberechnung bei der Kataraktchirurgie für das pseudophake Auge mit einfachen Mitteln abgeschätzt werden kann. Dieses Verfahren basiert generell auf einer Linsenberechnung mit Vergenzformeln (z. B. Haigis‑, Hoffer-Q- oder Castrop-Formel) und beschränkt sich auf lineare Gauß-Optik.

## Bestimmung der Vergenzen

**Figure 1:**
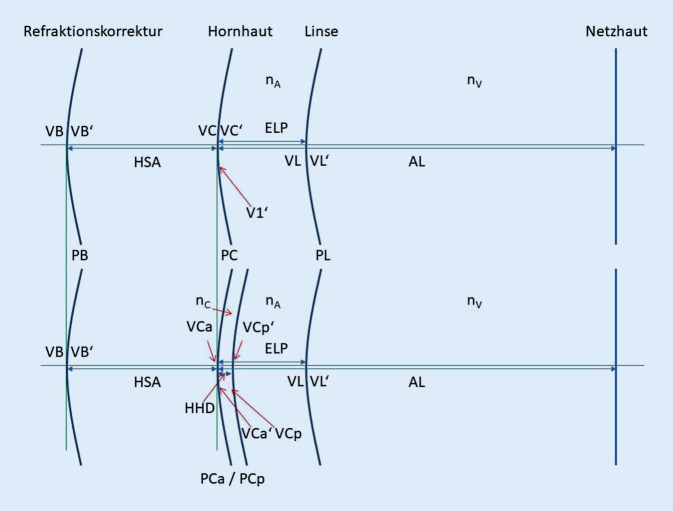

Nahezu alle heute offengelegten Vergenzformeln zur Berechnung des Brechwertes einer Intraokularlinse basieren auf einem vereinfachten pseudophaken Augenmodell mit 3 oder 4 refraktiven Grenzflächen [8, 10]: Die postoperative Brillenkorrektur oder Zielrefraktion (PB) berücksichtigt in der Brillenebene die Hornhaut, die entweder durch das Modell einer „dünnen Linse“ (PC, z. B. bei der Haigis-Formel) oder einer „dicken Linse“ (PCa/PCp, z. B. bei der Castrop-Formel) beschrieben wird, sowie die zu implantierende Kunstlinse (PL). Die Abb. 1 stellt das Schema des pseudophaken Augenmodells für die Berechnung der Kunstlinsenstärke dar. Im oberen Teil wird die Hornhaut als „dünne Linse“ und im unteren Teil als „dicke Linse“ berücksichtigt.

**Figure 2:**
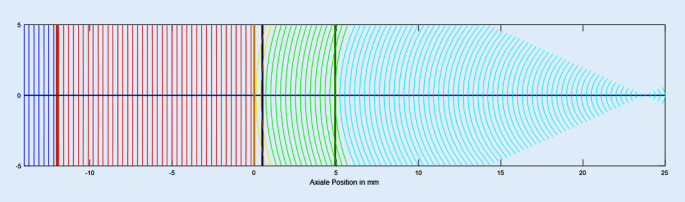

Die Vergenz beschreibt die Krümmung der Wellenfront an einer bestimmten axialen Position im Auge (siehe Abb. 2) [13]. Bei theoretisch-optischen Formeln, basierend auf einer Vergenztransformation, wird, ausgehend von einer Objektebene im Unendlichen (Objektentfernung o = ∞, Fernsicht) oder im Endlichen (Objekt z. B. in 5 m vor der Brillenkorrektur, o = 5 m), eine Vergenz sukzessive durch alle optischen Medien und Grenzflächen verfolgt [7, 8, 10] und so die Vergenz (VL) in der Ebene der Linse ermittelt. Dann wird die Vergenz hinter der Linse (VL′) berechnet, die benötigt wird, um eine scharfe Abbildung auf der Netzhaut zu erreichen. Der Vergleich der beiden Vergenzen hinter und vor der Intraokularlinse (Differenz aus VL′ und VL) ergibt den Brechwert der korrigierenden Linse PL. Für das pseudophake Augenmodell mit 3 refraktiven Grenzflächen (z. B. Haigis-Formel oder Hoffer-Q-Formel) gilt:

1 $$\begin{array}{l} \mathrm{VB}=0(\text{Objekt im Unendlichen})\\ \text{ oder }\\ \mathrm{VB}=-1/\mathrm{o}\\ \mathrm{VB'}=\mathrm{VB}+\mathrm{PB}\\ \begin{array}{l} \mathrm{VC}=\mathrm{VB'}/(1-\mathrm{VB'}\cdot \mathrm{HSA})\\ \begin{array}{l} \mathrm{VC'}=\mathrm{VC}+\mathrm{PC}\\ \mathrm{VL}=\mathrm{VC'}/(1-\mathrm{VC'}\cdot \mathrm{ELP}/\mathrm{n}_{\mathrm{A}})\\ \mathrm{VL'}=\mathrm{n}_{\mathrm{V}}/(\mathrm{AL}-\mathrm{ELP}) \end{array} \end{array} \end{array}$$

Für das pseudophake Augenmodell mit 4 refraktiven Grenzflächen gilt:

2 $$\begin{array}{l} \mathrm{VB}=0(\text{Objekt im Unendlichen})\\ \text{oder}\\ \mathrm{VB}=-1/\mathrm{o}\\ \mathrm{VB'}=\mathrm{VB}+\mathrm{PB}\\ \begin{array}{l} \mathrm{VC}=\mathrm{VB'}/(1-\mathrm{VB'}\cdot \mathrm{HSA})\\ \mathrm{VC}_{\mathrm{a}}\mathrm{'}=\mathrm{VC}+\mathrm{PC}_{\mathrm{a}}\\ \begin{array}{l} \mathrm{VC}_{\mathrm{p}}=\mathrm{VC}_{\mathrm{a}}\mathrm{'}/(1-\mathrm{VC}_{\mathrm{a}}\mathrm{'}\cdot \mathrm{HHD}/\mathrm{n}_{\mathrm{C}})\\ \mathrm{VC}_{\mathrm{p}}\mathrm{'}=\mathrm{VC}_{\mathrm{p}}+\mathrm{PC}_{\mathrm{p}}\\ \begin{array}{l} \mathrm{VL}=\mathrm{VC}_{\mathrm{p}}\mathrm{'}/(1-\mathrm{VC}_{\mathrm{p}}\mathrm{'} \\ \quad \cdot (\mathrm{ELP}-\mathrm{HHD})/\mathrm{n}_{\mathrm{A}})\\ \mathrm{VL'}=\mathrm{n}_{\mathrm{V}}/(\mathrm{AL}-\mathrm{ELP}) \end{array} \end{array} \end{array} \end{array}$$

### Abbildungsmaßstab bei klassischen Vergenzformeln

Exemplarisch sollen hier die Haigis-Formel, die Hoffer-Q-Formel und die Castrop-Formel, untersucht für die Vorhersage des Abbildungsmaßstabs, untersucht werden. Für Objekte in o = ∞ berechnet sich der Abbildungsmaßstab aus dem Verhältnis der retinalen Bildgröße zum Winkel unter dem ein Objekt wahrgenommen wird, ausgedrückt im Bogenmaß (Einheit: m). Dagegen berechnet sich der Abbildungsmaßstab für Objekte im Endlichen (z. B. o = 5 m) direkt aus dem Verhältnis von retinaler Bildgröße zur Objektgröße (dimensionslos). Die einfachste Möglichkeit der Berechnung des Abbildungsmaßstabs M_∞3/4_ erhält man für Objekte im Unendlichen, indem man die das Produkt aller Vergenzen unmittelbar vor refraktiven Grenzflächen (mit Ausnahme von VB, da VB = 0) durch das Produkt aller Vergenzen direkt nach refraktiven Grenzflächen dividiert. Für das Modell mit 3/4 Grenzflächen bedeutet das

3 $$M_{\mathrm{\infty }3}=\frac{\mathrm{VC}\cdot \mathrm{VL}}{\mathrm{V}B^{\mathrm{'}}\cdot \mathrm{V}C^{\mathrm{'}}\cdot \mathrm{V}L^{\mathrm{'}}}$$

4 $$M_{\mathrm{\infty }4}=\frac{\mathrm{VC}_{a}\cdot \mathrm{VC}_{p}\cdot \mathrm{VL}}{\mathrm{V}B^{\mathrm{'}}\cdot \mathrm{VC}_{a}\mathrm{'}\cdot \mathrm{VC}_{p}\mathrm{'}\cdot \mathrm{V}L^{\mathrm{'}}}$$

Für Objekte im Endlichen (VB ≠ 0) berechnet sich der Abbildungsmaßstabs M_3/4_ aus dem Verhältnis aller Vergenzen vor refraktiven Grenzflächen durch das Produkt aller Vergenzen nach refraktiven Grenzflächen dividiert. Für das Modell mit 3/4 Grenzflächen bedeutet das

5 $$M_{3}=\frac{VB\cdot VC\cdot VL}{VB^{'}\cdot VC^{'}\cdot VL^{'}}$$

6 $$M_{4}=\frac{VB\cdot VC_{a}\cdot VC_{p}\cdot VL}{VB^{'}\cdot VC_{a}'\cdot VC_{p}'\cdot VL^{'}}$$

Die Haigis-Formel ist charakterisiert durch ein pseudophakes Augenmodell mit 3 refraktiven Grenzflächen (s. Abb. 1 oben). Bei der Haigis-Formel leitet sich der Brechwert der Hornhaut aus dem Krümmungsradius der Vorderfläche R_a_ ab mit PC = (1,3315 − 1,00)/R_a_. Die effektive Linsenposition ELP [13, 14] berechnet sich zu ELP = a0 + a1·ACD + a2 AL, wenn ACD und AL die Augenlänge sowie die phake Vorderkammertiefe bezeichnen. Mit den Brechungsindizes für Kammerwasser und Glaskörper n_A_ = n_V_ = 1,336 und dem Hornhautscheitelabstand HSA = 12 mm, der den Abstand zwischen dem hinteren Scheitel der Brillenkorrektur und dem Hornhautscheitel beschreibt, und Gln. 1 sowie Gl. 3 bzw. 5 kann man direkt den Abbildungsmaßstab für Objekte im Unendlichen/Endlichen ermitteln.

Die Hoffer-Q-Formel ist ebenfalls charakterisiert durch ein pseudophakes Augenmodell mit 3 refraktiven Grenzflächen (s. Abb. 1 oben). Bei der Hoffer-Q-Formel [3, 4, 18] wird zunächst die Augenlänge nach oben begrenzt auf 31 mm sowie nach unten auf 18,5 mm. Der Brechwert der Hornhaut ergibt sich aus dem Krümmungsradius der Vorderfläche R_a_ zu PC = (1,3375 − 1,00)/R_a_. Die Berechnung der effektiven Linsenposition ELP ist in der Veröffentlichung [4, 18] im Detail beschrieben. Mit n_A_ = n_V_ = 1,336 und HSA = 12 mm und Gln. 1 sowie Gl. 3 bzw. 5 kann man direkt den Abbildungsmaßstab für Objekte im Unendlichen/Endlichen ermitteln.

Die Castrop-Formel ist beschrieben durch ein pseudophakes Augenmodell mit 4 refraktiven Grenzflächen (s. Abb. 1 unten) und 3 Formelkonstanten C, H und R. Der Korrekturwert R wird auf die vorhergesagte Refraktion in der Brillenebene aufaddiert. Der Brechwert der Hornhautvorderfläche ergibt sich aus dem Krümmungsradius der Vorderfläche R_a_ zu PC_a_ = (1,376 − 1,00)/R_a_ und der Brechwert der Rückfläche PC_p_ aus der Hornhautrückflächenkrümmung R_p_ zu PC_p_ = (1,376 − 1,336)/R_p_. Die Berechnung der effektiven Linsenposition ELP erfolgt aus der phaken Vorderkammertiefe ACD und Linsendicke LT mit ELP = ACD + C·LD + H. Mit n_A_ = n_V_ = 1,336 und HSA = 12 mm und Gln. 2 sowie Gl. 4 bzw. 6 kann man direkt den Abbildungsmaßstab für Objekte im Unendlichen/Endlichen ermitteln.

## Ergebnisse

Die Anwendung dieses einfachen Verfahrens zur Abschätzung des Abbildungsmaßstabs des (brillenkorrigierten) pseudophaken Auges nach der Kataraktoperation aus den biometrischen Daten sowie der intendierten Refraktion auf Brillenebene soll anhand beider Augen von 2 Beispielen gezeigt werden.

Beispiel 1 illustriert die klinisch nicht seltene Situation, dass das zweite Auge auf Emmetropie operiert werden soll, am ersten Auge aber postoperativ eine Ametropie vorliegt (refraktive Aniseikonie).

Beispiel 2 zeigt die ebenfalls gängige Situation einer Anisomyopie. Hier gilt es, zwischen Aniseikonie und Anisometropie abzuwägen und einen refraktiven Kompromiss zu finden (axiale Aniseikonie).

**Figure 3:**
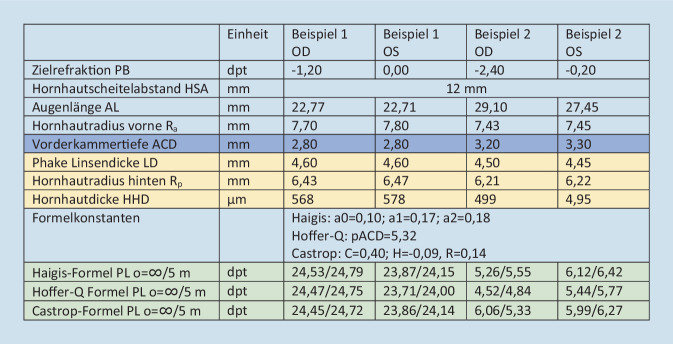

Die den Beispielen zugrunde gelegten biometrischen Daten sind in Abb. 3 zusammengefasst. Für die Hoffer-Q-Formel werden neben der Formelkonstante („personalized ACD“ [pACD]) ausschließlich die Augenlänge AL sowie der Radius der Hornhautvorderfläche R_a_ benötigt. Für die Haigis-Formel wird neben dem Konstanten-Triplet a0/a1/a2, der AL und R_a_ die phake Vorderkammertiefe ACD zur Abschätzung der effektiven Linsenposition herangezogen. Die Castrop-Formel interpretiert die Hornhaut als „dicke“ Meniskuslinse und greift auf den Krümmungsradius beider Hornhautflächen zu. Daneben werden 3 Formelkonstanten (C, H und R) sowie AL, ACD, die phake Linsendicke LD sowie die Hornhautdicke HHD in die Berechnung einbezogen.

In Tab. 1 sind die geschätzten Linsenpositionen (ELP) sowie die resultierenden Brechwerte der Linsen (PL) für die 4 Augen (2 Beispiele) jeweils berechnet mit den 3 Formeln und für Objekte im Unendlichen sowie Objekte in einem Objektabstand von 5 m protokolliert. Daneben sind in Tab. 1 die jeweiligen Vergenzen vor und hinter den 3 (Haigis-Formel und Hoffer-Q-Formel) bzw. 4 (Castrop-Formel) refraktiven Grenzflächen notiert, die aus den Gln. 1 und 2 berechnet wurden. Diese Vergenzen werden in einem weiteren Schritt herangezogen, um mit den Gln. 3 und 4 bzw. den Gln. 5 und 6 den Abbildungsmaßstab des pseudophaken Auges für ein pseudophakes Augenmodell mit 3 bzw. 4 refraktiven Grenzflächen mit einem Objekt im Unendlichen bzw. in einer endlichen Entfernung abzuschätzen. Je nach Implementierung in einem Softwaremodul sind die Vergenzen vor und hinter den Grenzflächen als Zwischenschritte der Berechnung direkt abrufbar oder können sehr einfach bereitgestellt werden.

**Table 1:** 

Formel	Objektabstand	ELP/Brechwert; Vergenz	Beispiel 1 OD	Beispiel 1 OS	Beispiel 2 OD	Beispiel 2 OS
Haigis-Formel	o = ∞	ELP in mm/PL in dpt	4,7/24,52	4,7/23,87	5,8/5,26	5,6/6,12
		VB/VB′	0/−1,20	0/0	0/−2,40	0/−0,20
		VC/VC′	−1,18/41,93	0/42,56	−2,33/42,84	−0,20/44,97
		VL/VL′	49,16/73,69	50,01/73,88	52,71/57,97	55,36/61,48
	o = 5 m	ELP in mm/PL in dpt	4,7/24,79	4,7/24,15	5,8/5,55	5,6/6,42
		VB/VB′	−0,20/−1,40	−0,20/−0,20	−0,20/−2,60	−0,20/−0,40
		VC/VC′	−1,38/41,74	−0,20/42,36	−2,52/42,65	−0,40/44,77
		VL/VL′	48,89/73,69	49,73/73,88	52,43/57,98	55,06/61,48
Hoffer-Q-Formel	o = ∞	ELP in mm/PL in dpt	4,9/24,47	4,9/23,71	6,5/4,52	6,2/5,44
		VB/VB′	0/−1,2	0/0	0/−2,40	0/−0,20
		VC/VC′	−1,18/42,65	0/43,27	−2,33/43,59	−0,20/45,72
		VL/VL′	50,93/75,41	51,72/75,43	56,28/60,80	58,93/64,37
	o = 5 m	ELP in mm/PL in dpt	4,9/24,75	4,9/24,00	6,5/4,84	6,2/5,77
		VB/VB′	−0,2/−1,40	−0,20/−0,20	−0,20/−2,60	−0,20/−0,40
		VC/VC′	−1,38/42,45	−0,20/43,07	−2,52/43,30	−0,40/45,52
		VL/VL′	50,66/75,41	51,43/75,43	55,96/60,80	58,60/64,37
Castrop-Formel	o = ∞	ELP in mm/PL in dpt	4,6/24,45	4,6/23,86	4,9/5,06	5,0/5,99
		VB/VB′	0/−1,06	0/0,14	0/−2,26	0/−0,06
		VC_a_/VC_a_′	−1,05/47,78	0,14/48,35	−2,20/48,96	−0,06/51,10
		VC_p_/VC_p_′	48,75/42,53	49,35/43,17	49,84/43,40	52,05/45,62
		VL/VL′	48,70/73,15	49,52/73,38	50,65/55,72	53,94/59,92
	o = 5 m	ELP in mm/PL in dpt	4,6/24,72	4,6/24,14	4,9/5,33	5,0/6,27
		VB/VB′	−0,2/−1,26	−0,20/−0,06	−0,20/−2,46	−0,20/−0,26
		VC_a_/VC_a_′	−1,24/47,59	−0,06/48,15	−2,39/48,77	−0,26/50,90
		VC_p_/VC_p_′	48,54/42,32	49,14/42,96	49,65/43,20	51,85/45,42
		VL/VL′	48,43/73,15	49,25/73,38	50,39/55,72	53,65/59,92

Die Ergebnisse für den Abbildungsmaßstab M sind in Tab. 2 zusammengetragen. Liegt die Objektebene im Unendlichen, so beschreibt der mit den Beziehungen 3 und 4 ermittelte Abbildungsmaßstab M_∞_. das Verhältnis aus retinaler Bildgröße zum Eingangswinkel unter dem ein Objekt wahrgenommen wird. So wird in Beispiel 1 am rechten Auge (OD) mit der Haigis-Formel/Hoffer-Q-Formel/Castrop-Formel ein Abbildungsmaßstab von M_∞_ = 0,015683 m/0,01561 m/0,01577 m ermittelt. Das bedeutet dass ein Objekt, das unter einem Winkel von 1 Bogenminute entsprechend einer Sehschärfe von 100 % abgebildet wird (z. B. Öffnung eines Landolt-Ringes) auf der Netzhaut eine Bildgröße von 4,562/4,542/4,587 µm aufweist.

**Table 2:** 

Abbildungsmaßstab M × 1000	Objektabstand	Beispiel 1 OD	Beispiel 1 OS	Beispiel 2 OD	Beispiel 2 OS
Haigis-Formel	o = ∞ (m)	M_∞3_ = 15,6833	M_∞3_ = 15,9028	M_∞3_ = 20,6302	M_∞3_ = 19,9740
	Relativ in %	1,3898		−3,2318	
	o = 5 m (1)	M_3_ = −3,1268	M_3_ = −3,1703	M_3_ = −4,1123	M_3_ = −3,9812
	Relativ in %	1,3840		−3,2385	
Hoffer-Q-Formel	o = ∞(m)	M_∞3_ = 15,6130	M_∞3_ = 15,8459	M_∞3_ = 20,6420	M_∞3_ = 19,9752
	Relativ in %	1,4809		−3,2838	
	o = 5 m (1)	M_3_ = −3,1125	M_3_ = −3,1588	M_3_ = −4,1136	M_3_ = −3,9805
	Relativ in %	1,4759		−3,2894	
Castrop-Formel	o = ∞(m)	M_∞4_ = 15,7686	M_∞4_ = 15,9842	M_∞4_ = 20,7635	M_∞4_ = 20,0846
	Relativ in %	1,3578		−3,3243	
	o = 5 m (1)	M_4_ = −3,1438	M_4_ = −3,1866	M_4_ = −4,1396	M_4_ = −4,0037
	Relativ in %	1,3519		−3,3378	

Verwendet man dagegen eine Sehzeichentafel für die Visusstufe 1,0 in einem Abstand von 5 m (gemäß ISO-Norm ist eine Sehschärfeprüfung mit einer Messstrecke von 4–6 m vorgesehen) und die Berechnung des Abbildungsmaßstabs anhand der Formelbeziehungen 5 und 6, so kann die Größe der Öffnung des Normsehzeichens (z. B. Landolt-Ring) in der Projektionsebene (5 m) über die retinale Bildgröße (z. B. 4,56 µm) und den Abbildungsmaßstab M. in Tab. 2 abgeschätzt werden. Anhand von Beispiel 1 erhält man hier für das rechte Auge mit der Haigis-Formel/Hoffer-Q-Formel/Castrop-Formel einen Abbildungsmaßstab von M = −0,00313/−0,00311/−0,00314 und für eine retinale Bildgröße von 4,56 µm die Öffnung des Normsehzeichens in einem Abstand von o = 5 mit 1,458/1,465/1,450 mm. Das negative Vorzeichen bei M. deutet an, dass das retinale Bild gegenüber dem Objekt invertiert ist.

Vergleicht man die retinale Bildgröße beider Augen für Beispiel 1 für die Haigis/Hoffer-Q/Castrop-Formel für einen Objektabstand von 5 m miteinander, so ergibt sich ein relativer Bildgrößenunterschied derart, dass das linke Auge (Brillenrefraktion plan) gegenüber dem rechten Auge (Brillenrefraktion −1,2 dpt) mit dem in Abb. 3 angegebenen Linsenbrechwert 1,38 %/1,47 %/1,35 % vergrößert abbildet. Für Beispiel 2 bildet entsprechend das rechte Auge (mit einer Brillenrefraktion von −2,4 dpt) mit dem in Abb. 3 angegebenen Linsenbrechwert um 3,24 %/3,29 %/3,34 % gegenüber dem linken Auge (mit einer Brillenrefraktion von −0,2 dpt) vergrößert ab.

## Diskussion

Eine überwiegende Mehrheit der Berechnungskonzepte für Intraokularlinsenbrechwerte basieren auf Vergenzen. Dabei wird für eine Objektentfernung im Unendlichen o = ∞ bzw. Objektentfernungen im Endlichen (z. B. Messdistanz bei der Refraktometrie mit o = 5 m) die Eingangsvergenz in der Brillenebene berechnet (VB = 0 bzw. VB = −1/o). Anschließend wird sukzessive in der Ebene jeder refraktiven Grenzfläche die Vergenz um den jeweiligen Brechwert korrigiert und beim Durchlaufen eines optischen Mediums mit der Dicke d und dem Brechungsindex *n* die Vergenz V. nach der klassischen Vergenzformel V./(1‑V.·d/*n*) transformiert. Diese Vergenztransformation ist z. B. aus der Umrechnung einer Brillenrefraktion in eine Kontaktlinsenrefraktion in der Ophthalmologie allgemein gebräuchlich. Die Vergenz, die hinter der Linse benötigt wird, um scharf auf die Netzhaut abzubilden, berechnet sich aus n_V_/(AL-ELP), wenn n_V_ den Brechungsindex des Glaskörpers und AL-ELP die Glaskörperstrecke hinter der Linse beschreibt. Die (dünne) Intraokularlinse muss nun das Vergenzdefizit (Vergenz hinter der Linse abzüglich der Vergenz vor der Linse) ausgleichen [8].

Abgesehen von rein empirischen Formeln (Regressionsformeln oder Verfahren des maschinellen Lernens) und Raytracingansätzen („full aperture raytracing“) machen sich quasi alle theoretisch optischen Formeln diese einfachen Zusammenhänge der linearen Gauß-Optik mit ihrer Vereinfachung auf den paraxialen Raum zunutze. Die Variation der heute verfügbaren Berechnungskonzepte bezieht sich in der Hauptsache auf die unterschiedliche Interpretation der biometrischen Messgrößen bei der Einarbeitung in das pseudophake Augenmodell, in der Hauptsache die Abschätzung der Linsenposition im pseudophaken Auge auf der Basis der biometrischen Größen des phaken Auges sowie verschiedener Interpretationen (z. B. Hornhautbrechwert aus dem Krümmungsradius der Vorderfläche) oder Korrekturwerte („fudge factors“). Je nachdem wie die Formel in einem Softwaremodul implementiert wurde, liegen die Vergenzen unmittelbar vor und hinter den refraktiven Grenzflächen des pseudophaken Modells unmittelbar vor, sodass sie direkt für die Abschätzung des retinalen Abbildungsmaßstabs herangezogen werden können. Liegen diese Zwischenschritte nicht vor, so stellt eine entsprechende Erweiterung der Programmierung keinen großen Aufwand dar.

In den vergangenen Jahren wurden große Anstrengungen unternommen, die Biometrie weiter zu verbessern und die Linsenberechnung weiter zu optimieren, sodass heute bei einer Kataraktoperation unter „Standardbedingungen“ ein mittlerer absoluter Vorhersagefehler in der Refraktion von 0,3–0,4 dpt durchaus zu erwarten ist, dabei liegen etwa 65–80 % der Refraktionsergebnisse in einem Bereich von ±0,5 dpt um die avisierte Zielrefraktion. Diese Fortschritte in der Biometrie und der Linsenberechnung waren das Fundament für moderne Linsenkategorien. wie z. B. Enhanced-Depth-Of-Focus(EDOF)-Linsen, die nur dann sinnvoll eingesetzt werden können, wenn die Zielrefraktion auch tatsächlich erreicht wird.

Die retinale Bildgröße bzw. die Aniseikonie als den retinalen Bildgrößenunterschied beider Augen hat man bei der Suche nach der perfekten Refraktion und der Verbesserung der Abbildungseigenschaft durch neue Optikkonzepte (z. B. aberrationskorrigierende Linsen) aus den Augen verloren. In den meisten Fällen sind Augen isometrisch aufgebaut, die biometrischen Messgrößen sowie die Refraktion beider Augen weichen nicht wesentlich voneinander ab. So liegt die Aniseikonie beim phaken Auge in der Regel bei Werten unterhalb von 0,5 %. Ab einer Aniseikonie von 0,75 % können erste Symptome auftreten (Wilkonson und Shahid, persönliche Kommunikation), und in manchen Fällen wird ein Bildgrößenunterschied von 5 % oder mehr gemessen. Für pseudophake Augen liegen nur sehr dürftige Studien vor über die Prävalenz oder Ausprägung von Aniseikonien [21]. Allerdings kann man davon ausgehen, dass die Problematik durch die Kataraktoperation eher verschärft als vermindert wird. Und gerade für Patienten, bei denen nur 1 Auge zur Kataraktoperation vorgesehen ist (z. B. traumatische Katarakt) oder ein großes Zeitintervall geplant ist zwischen den Operationen beider Augen, sollten auch die optischen Bedingungen nach dem Eingriff an einem Auge berücksichtigt werden.

Generell können Aniseikonien in Form eines Bildgrößenunterschiedes beider Augen zu Übelkeit, Kopfschmerzen, Einschränkung oder Verlust des Stereosehens bis hin zur Desorientierung, Störungen des Gleichgewichtssinns oder Suppression führen. Die Literatur beschreibt, dass maximal 2–5 % Bildgrößenunterschied beider Augen für einen kurzen Zeitraum möglicherweise toleriert werden, auf Dauer treten bei Bildgrößenunterschieden von 2–3 % jedoch bereits erhebliche Fusionsprobleme auf [19]. Für asthenopische Beschwerden liegt die Toleranzschwelle womöglich noch deutlich geringer, allerdings wird eine erhebliche Streuung in der Bevölkerung vermutet [5–7, 9, 11, 15, 16, 19, 20]. Für pseudophake Augen sind hier keine gesicherten Literaturdaten bekannt, und speziell im Hinblick auf moderne EDOF oder Multifokallinsen wären Untersuchungen zur Toleranz von Bildgrößenunterschieden essenziell.

Bei der Biometrie und Linsenberechnung im Vorfeld einer Kataraktoperation sind alle für die Abschätzung des Abbildungsmaßstabs beider Augen notwendigen Messgrößen verfügbar. So kann, speziell wenn mit vergenzbasierten Formeln wie der Haigis‑, Hoffer-Q-, Olsen‑, Castrop-Formel oder auch vielen anderen Berechnungskonzepten gearbeitet wird, immer dann, wenn die effektive Linsenposition ELP im Berechnungskonzept explizit abgeschätzt wird [13, 14], mit einer sehr einfachen Zusatzberechnung der Abbildungsmaßstab für Objekte im Unendlichen oder für Objekte in einer Refraktionsmessdistanz berechnet werden. Da in der Regel beide Augen bei der Biometrie gemessen werden, kann aus dem Vergleich des Abbildungsmaßstabs beider Augen die Aniseikonie nach der Kataraktoperation ermittelt werden. Dass die Ergebnisse im Vergleich der 3 hier untersuchten Formeln geringe Unterschiede aufweisen (s. Tab. 2), ist von untergeordneter Bedeutung, sofern man für die Beurteilung der zu erwartenden Aniseikonie das gleiche Berechnungskonzept (auf der Basis derselben Formel) heranzieht.

Entsprechend können dann, wenn postoperativ mit einer klinisch relevanten Aniseikonie zu rechnen ist, entsprechende Gegenmaßnahmen eingeleitet werden. Kennt man die Biometriedaten, dann kann im Einzelfall durch eine Modifikation der Zielrefraktion und einen Ausgleich mit einer Brillen- oder Kontaktlinsenkorrektur [21], durch eine einzeitige oder zweizeitige Kombination der Kataraktoperation mit einem hornhautchirurgischen Eingriff, eine Kombination der Kapselsacklinse mit einer Add-on-Linse [8] oder auch durch die Wahl eines geeigneten Linsendesigns gegengesteuert werden. So kann z. B. aus der Bandbreite der kommerziell verfügbaren Intraokularlinsenmodelle mit der Auswahl eines Optikmaterials (Brechungsindex bzw. zentrale Linsendicke) oder des Coddington-Shape-Faktors der Abbildungsmaßstab im Sinne eines eikonischen Linsendesigns in Grenzen variiert werden. Eine deutlich größere Variationsmöglichkeit steht selbstredend mit einer Kombination aus postoperativer Brillenkorrektur und entsprechender Anpassung der Linsenstärke zur Verfügung, was allerdings mit dem Patienten im Vorfeld abgeklärt werden sollte.

Einschränkend sei angemerkt, dass die physikalisch-optische Aniseikonie nicht notwendigerweise mit der subjektiv empfundenen identisch sein muss. So kann abhängig von der Augenlänge die Dichte der retinalen Photorezeptoren im Bereich der Fovea variieren. Bei Anisometropie kann zudem die unterschiedliche Brillenkorrektion die Aniseikonie erheblich beeinflussen.

*Schlussfolgernd* soll diese Arbeit daran erinnern, dass die retinale Bildgröße bzw. der Abbildungsmaßstab als mögliche Ursache für asthenopische Beschwerden stets ins Kalkül gezogen werden sollte. Bei der Planung einer Kataraktoperation liegen speziell bei vergenzbasierten (sog. theoretisch optischen) Formeln alle Größen vor, die für eine Abschätzung der retinalen Bildgröße sowie des Abbildungsmaßstabs nach der Kataraktoperation mit Implantation einer Kunstlinse benötigt werden. Da in aller Regel die biometrische Vermessung an beiden Augen durchgeführt wird, sollte der Abbildungsmaßstab für beide Augen ermittelt werden, sodass der Vergleich der Netzhautbildgrößen ein Maß für die zu erwartende Aniseikonie nach der Kataraktoperation liefert. Das hier vorgestellte Berechnungskonzept wurde exemplarisch für 3 gängige Linsenberechnungsformeln gezeigt, es kann aber sehr einfach auch auf andere Berechnungsformeln erweitert werden, sofern explizit die axiale Position der Kunstlinse im Auge und der Hornhaut- und der Brechwert der für die Implantation vorgesehenen Kunstlinse bekannt sind.

## References

[1] Fyodorov SN, Galin MA, Linksz A (1975). Calculation of the optical power of intrao-cular lenses. Invest Ophthalmol.

[2] Gernet H, Ostholt H, Werner H (1970). Die präoperative Berechnung intraocularer Binkhorst-Linsen.

[3] Hoffer KJ (1980). Steps for IOL power calculation. Am Intraocul Implant Soc.

[4] Hoffer KJ (1993). The Hoffer Q formula: a comparison of theoretic and regression formulas. J Cataract Refract Surg.

[5] Katsumi O, Tanino T, Hirose T (1986). Effect of aniseikonia on binocular function. Invest Ophthalmol Vis Sci.

[6] Krzizok T, Kaufmann H, Schwerdtfeger G (1996). Binokulare Probleme durch Aniseikonie und Anisophorie nach Katarakt-Operation. Klin Monbl Augenheilkd.

[7] Langenbucher A, Reese S, Huber S, Seitz B (2005). Compensation of aniseikonia with toric intraocular lenses and spherocylindrical spectacles. Ophthalmic Physiol Opt.

[8] Langenbucher A, Szentmáry N, Seitz B (2007). Magnification and accommodation with phakic intraocular lenses. Ophthalmic Physiol Opt.

[9] Langenbucher A, Szentmáry N (2008). Anisometropie und Aniseikonie – ungelöste Probleme der Kataraktchirurgie. Klin Monbl Augenheilkd.

[10] Langenbucher A, Viestenz A, Seitz B, Brünner H (2007). Computerized calculation scheme for retinal image size after implantation of toric intraocular lenses. Acta Ophthalmol Scand.

[11] Lubkin V, Shippman S, Bennett G, Meininger D, Kramer P, Poppinga P (1999). Aniseikonia quantification: error rate of rule of thumb estimation. Binocul Vis Strabismus Q.

[12] Melles RB, Kane JX, Olsen T, Chang WJ (2019). Update on intraocular lens calculation formulae. Ophthalmology.

[13] Olsen T, Hoffmann P (2014). C constant: new concept for ray tracing-assisted intraocular lens power calculation. J Cataract Refract Surg.

[14] Olsen T (2007). Calculation of intraocular lens power: a review. Acta Ophthalmol Scand.

[15] Pittke EC (1987). Fusionsbreite und Aniseikonie. Experimentelle Untersuchungen zur Aniseikonietoleranz bei einseitiger Aphakie. Klin Monbl Augenheilkd.

[16] Rabin J, Bradley A, Freeman RD (1983). On the relation between aniseikonia and axial anisometropia. Am J Optom Physiol Opt.

[17] Savini G, Taroni L, Hoffer KJ (2020). Recent developments in intraocular lens power calculation methods-update 2020. Ann Transl Med.

[18] Shammas HJ (2004). Intraocular lens power calculations.

[19] South J, Gao T, Collins A, Turuwhenua J, Robertson K, Black J (2019). Aniseikonia and anisometropia: implications for suppression and amblyopia. Clin Exp Optom.

[20] Westheimer G (2007). First description of aniseikonia. Br J Ophthalmol.

[21] Winn B, Ackerley RG, Brown CA, Murray FK, Prais J, St John MF (1988). Reduced aniseikonia in axial anisometropia with contact lens correction. Ophthalmic Physiol Opt.

